# Editorial: Innovators in analytical chemistry

**DOI:** 10.3389/fchem.2023.1149382

**Published:** 2023-02-08

**Authors:** Camelia Bala, Nicole Jaffrezic-Renault, Gabriella Massolini, Giovanni Valenti

**Affiliations:** ^1^ University of Bucarest, Bucarest, Romania; ^2^ University Claude Bernard Lyon 1, Lyon, France; ^3^ University of Pavia, Pavia, France; ^4^ University of Bologna, Bologna, Italy

**Keywords:** analytical chemistry and techniques, mass spectrometry, laser-induced breakdown spectroscopy (LIBS), reverse transcription recombinase polymerase amplification, biological applications, environmental applications

This Research Topic is devoted to innovations in Analytical Chemistry. Three articles and one mini review were published.

Mass spectrometry is the basic technique for two papers. Matrix-assisted Laser Desorption Ionization Mass Spectrometry Imaging (MALDI-MSI) coupled with other imaging modalities in multimodal approaches is reviewed for *in vivo* and *in vitro* biological applications. (Tuck et al.) Differential cell-surface N-glycosylation of ovarian cancer SKOVS cells were analyzed by HPLC-MS/MS on the enriched and labeled N-glycopeptides and compared to non-cancerous ovarian epithelial IOSE80 cell lines. This study provides important N-glycoprotein biomarker candidates for future studies (Zhou et al.).

Isothermal reverse transcription recombinase polymerase amplification (RT-RPA) is a simple, sensitive and cheap molecular diagnostic method. RT-RPA is here combined with a magnetic field-enhanced agglutination (MFEA) assay for the detection of the dengue virus (DENV). A rapid, sensitive and specific test is then obtained (Leon et al.).

